# Eating, feeding, and feeling: emotional responsiveness mediates longitudinal associations between maternal binge eating, feeding practices, and child weight

**DOI:** 10.1186/s12966-016-0415-5

**Published:** 2016-08-02

**Authors:** Jaclyn A. Saltzman, Maria Pineros-Leano, Janet M. Liechty, Kelly K. Bost, Barbara H. Fiese, the STRONG Kids Team

**Affiliations:** 1 Department of Human Development and Family Studies, University of Illinois at Urbana-Champaign, 904 South Nevada St., MC-081, Urbana, IL 61801 USA; 2 Illinois Transdisciplinary Obesity Prevention Program, University of Illinois, Champaign, USA; 3 School of Social Work, University of Illinois at Urbana-Champaign, 1010 West Nevada St., Urbana, IL USA; 4 College of Medicine, University of Illinois at Urbana-Champaign, 1010 West Nevada St., Urbana, IL 61801 USA; 5 Family Resiliency Center, University of Illinois at Urbana-Champaign, 904 South Nevada St., Urbana, IL 61801 USA

**Keywords:** Binge eating, Feeding practices, Intergenerational transmission, Parenting, Childhood obesity, Emotional responsiveness, Responsive parenting, Feeding Practices, Food-related parenting practices, Emotion regulation

## Abstract

**Background:**

Although it is known that maternal disordered eating is related to restrictive feeding practices, there is little research exploring mechanisms for this association or its effects on other feeding practices. The purpose of this study was to assess whether maternal emotion responses mediate the association between maternal binge eating (BE) and child feeding practices, in order to identify potential risk factors for feeding practices that influence child weight.

**Methods:**

This longitudinal observational study included (*n* = 260) mothers and children from the STRONG Kids Panel Survey. At Wave 1, children were an average of 37 months old (*SD* = 6.9), and at Wave 2 children were an average of 57 months old (*SD* = 8.3). Mothers self-reported their frequency of binge eating behavior (Wave 1), responses to children’s negative emotions (Wave 1), feeding practices (Wave 1 and Wave 2), and child height and weight were measured at both time points. Using bias-corrected bootstrapping procedures, we tested the hypothesis that longitudinal associations between maternal BE and nonresponsive parent feeding practices would be mediated by parents’ unsupportive responses to children’s negative emotion. We also tested a serial mediation model positing that maternal BE predicts child body mass index (BMI) percentile change 18-24 months later, indirectly through unsupportive responses to negative emotion and nonresponsive feeding practices.

**Results:**

Maternal BE predicted use of more nonresponsive feeding practices (e.g. Emotion Regulation, Restriction for Health, Pressure to Eat, and Food as Reward), indirectly through more Distress responses to children’s negative emotions. In the serial mediation model, maternal BE was associated with greater use of Distress responses, which indirectly predicted higher child BMI percentile through Food as Reward feeding practices.

**Conclusions:**

These results suggest that maternal eating and emotion responsiveness are important for understanding the interpersonal context of feeding behaviors, and child weight outcomes. Distress responses may serve as a risk factor for use of unhealthful feeding practices among mothers with BE and these responses may increase children’s risk for weight gain.

**Trial registration:**

This study used an observational prospective design. Therefore, it has not been registered as a clinical intervention trial.

**Electronic supplementary material:**

The online version of this article (doi:10.1186/s12966-016-0415-5) contains supplementary material, which is available to authorized users.

## Background

Childhood obesity is a risk factor for cardiovascular and metabolic disease later in life, and affects about 15 % of preschool-aged children in the United States [1, 2]. Childhood obesity is a complex problem that emerges as a result of the combined effects of multiple risk factors [3]. One risk factor for excessive weight gain in early childhood is the degree to which parents’ feeding practices are responsive to children’s cues of hunger and satiety [4–7]. Responsive feeding involves caregivers providing a variety of foods, setting up predictable routines around eating, and accepting when children report hunger or satiety, which protects children against excessive weight gain [6, 8, 9]. In contrast, non-responsive feeding involves patterns that include pressuring children to eat, monitoring and restricting the amount of food they eat, rather than respecting, responding, or listening to children’s cues of hunger and satiety, and may place children at risk for excessive weight gain. Responsive parenting generally describes patterns of parent-child interactions in which the parent attends to and responds appropriately to children’s cues of distress or pleasure, in a context characterized by routines and predictability for the child [8]. Responsiveness has most commonly been assessed in terms of responsiveness to children’s emotions [10–12], which is linked to child emotional understanding [13], and to more optimal emotion regulation [14–16]. However, given the substantial body of research on interconnections between emotion and eating behaviors [17], it is surprising that there has been so little research exploring emotional responsiveness in the context of feeding in the family system.

Conceptual scholarship has identified *parallels* in in how children develop emotion and energy-intake regulation behaviors in the context of the intergenerational transmission process [18]. Simply stated, maternal emotion regulation predicts child emotion regulation [11], and maternal energy-intake regulation predicts child energy-intake regulation [19–21]. Emotional responsiveness and feeding responsiveness, respectively, are socialization processes that mediate the association between maternal and child regulation behaviors [11, 18, 22]. Thus, maternal responsiveness may be a mechanism for the intergenerational transmission of self-regulation.

Most research exploring predictors of nonresponsive feeding practices has relied on maternal body mass index (BMI) as proxy for energy-intake regulation [6]. Although this link has laid important foundations, it must be noted that BMI does not always accurately reflect eating *behavior*. Research on maternal disordered eating and feeding responsiveness provides an avenue for examining how a behaviorally defined example of maternal energy-intake dysregulation is related to child outcomes. Mothers with eating disorders have children with more disordered eating behaviors than controls [23], however, the mechanisms for this transmission process are unclear. Promising evidence points to the potential that emotion regulation may serve as a mediator; recent empirical studies suggest that the intergenerational transmission of emotion and energy-intake dysregulation may not be parallel, but rather *intertwined* [24–26]. In order to disentangle these associations, further study is needed to elucidate how emotional and feeding responsiveness influence child weight outcomes. Therefore, the aim of this study was to examine whether and how emotional responsiveness influences feeding responsiveness and child weight outcomes, in the context of maternal binge eating (BE) behavior.

### Maternal binge eating, emotional, and feeding responsiveness

Binge eating (BE) describes eating substantially more than another person would in a similar situation, a single sitting, and within a relatively short amount of time, accompanied by feelings of distress and loss of control [27]. Binge eating is a form of self-regulatory dysfunction, and emerges from emotional and/or physiological distress. For example, BE can arise from a desire to escape from emotional distress, by serving as a maladaptive emotion regulation behavior that the individual expects to alleviate negative affect [28]. Binge eating can also develop as a result of restrained eating; when a restrained eater exceeds their desired daily energy-intake, their motivation to continue the diet is undermined, prompting a “what-the-heck” effect, in which the previously restrained individual then begins to eat whatever and however much they desire, often past the point of satiety [29–31].

Maternal BE has been associated with loss-of-control eating behaviors in children [20, 32], and feeding practices have been implicated as a putative mechanism for this association. In cross-sectional studies, maternal BE is related to use of restrictive feeding practices for children [26, 33, 34], which is particularly concerning given that restrictive feeding practices are strong predictors of increased child eating and weight gain [4, 25, 35, 36]. Longitudinal studies about the effects of maternal BE on feeding practices are scarce, so it is unclear whether maternal BE is related to non-responsive feeding generally, or restrictive feeding specifically.

Why would maternal BE and its caloric excess be associated with non-responsive feeding? People who engage in BE report using fewer emotion regulation strategies than healthy controls [37], and thus may have difficulty responding when children express negative emotions [38]. Emotional responsiveness describes how appropriate and sensitive a parent’s response is to a child’s display of emotion [39]. Distress responses to children’s negative emotions involve mothers’ focus on their own discomfort, anxiety, embarrassment, or other negative affect, rather than focus on alleviating the child’s negative emotions [10, 11, 40]. Feeding responsiveness and emotional responsiveness are part of the larger paradigm of responsive parenting [8]. Therefore, it is reasonable to suggest that emotional responsiveness may be particularly relevant for predicting feeding responsiveness among mothers who struggle with both emotion and energy-intake dysregulation. Nevertheless, to our knowledge, there are currently no studies that have assessed the longitudinal associations among maternal BE, emotional responsiveness, and feeding responsiveness. Longitudinal analyses allows researchers to identify pathways of risk, and to ascertain whether emotional responsiveness informs feeding responsiveness, or vice versa. Moreover, specific associations with other feeding practices have not yet been fully elucidated, limiting our understanding of how a range of possible feeding practices may be affected by maternal BE. Thus, in order to more fully understand how emotional and feeding responsiveness are related, it is necessary to establish how other feeding practices—such as giving children food as a reward, providing food to children to assuage negative emotions, or monitoring food intake—are related to emotional responsiveness.

### Emotional responsiveness, feeding responsiveness, and child weight outcomes

Child weight outcomes are the result of many interconnected and transactional influences across several domains [3], and have come under intense scrutiny in the last decade. Feeding practices are behaviors that parents use to provide nourishment to their children, and can range from parent provision of a balanced and varied array of foods, to parent restriction of intake of sugars and high-fat foods. These practices can generally be described as either responsive (e.g., attending to children’s internal cues of hunger/satiety), or non-responsive (e.g., providing external cues for children’s hunger/satiety), and are part of an overall framework of responsive parenting [6, 8, 9, 41]. Non-responsive feeding practices place children at risk for weight gain and obesity [5, 35].

Responsive parenting practices are also the foundation for parent-child attachment, by providing a secure base for children to rely on for emotional development and learning [42, 43]. Attachment theory casts caregivers as trusted models and guides for children to rely on for alleviating distress and providing security [44]. Emotions are monitored and regulated by caregiver behaviors and responses during infancy, but as children grow, they learn how to monitor and modify their own affect. By watching caregivers model appropriate emotion regulation behaviors, discuss affective states, and modify their environments to alleviate negative affect, children internalize their histories of interactions with caregivers, and develop expectations and scripts for interactions in the parent-child dyad [45]. Conceptual work drawing parallels between emotional and feeding responsiveness highlights responsiveness as a potentially modifiable risk factor for child energy-intake regulation [18]. Thus, it is important to assess whether emotional responsiveness is part of the foundation for feeding responsiveness, especially in the context of deficits in maternal energy-intake regulation.

### Current study

**Fig. 1:**
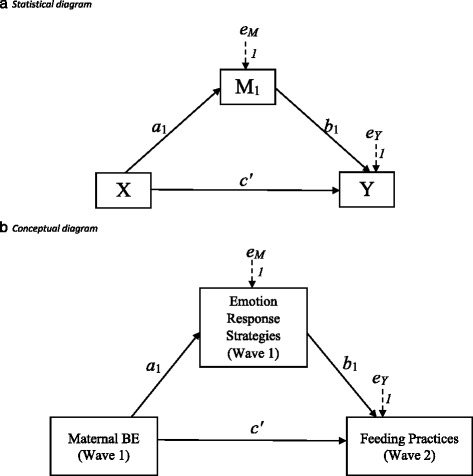
Statistical (Fig. 1a) and conceptual (Fig. 1b) diagrams of first hypothesis, in which maternal BE at Wave 1 (child age *M* = 37 months, *SD* = 6.94 months) predicts use of non-responsive feeding practices at Wave 2 (child age *M* = 57 months, *SD* = 8.32 months) directly (*c’*) and indirectly (*a*_*1*_ *b*_*1*_) through Emotion Response Strategies

**Fig. 2:**
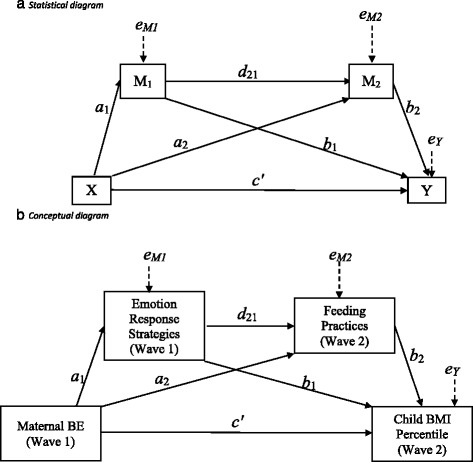
Statistical (Panel **a**) and conceptual (Panel **b**) diagrams of second hypothesis, in which maternal BE at Wave 1 (child age *M* = 37 months, *SD* = 6.94 months) predicts higher child BMI percentile at Wave 2 (child age *M* = 57 months, *SD* = 8.32 months) directly (*c’*), and indirectly through Emotion Response Strategies (a_1_b_1_), Feeding Practices (a_2_b_2_), and through both Emotion Responses and Feeding Practices (a_1_d_21_b_2_)

To our knowledge, this is one of the first studies to assess longitudinal associations between maternal binge eating, emotional responsiveness, and feeding responsiveness. There is also a dearth of literature linking maternal eating behaviors and feeding practices to child weight status, which if addressed could enhance understanding of potentially modifiable risk factors for childhood obesity. Therefore, the current study had two aims. First, we intend to assess whether maternal binge eating and responses to children’s negative emotions predicted self-reported responsive or non-responsive feeding practices among mothers of preschool aged children. We hypothesized that maternal BE at Wave 1 would be related to non-responsive feeding practices at Wave 2 directly, and indirectly through use of unsupportive responses to children’s negative emotions at Wave 1 (Fig. 1). Our second aim was to assess whether maternal BE (Wave 1), responses to children’s emotions (Wave 1), and feeding practices (Wave 2) predicted higher child BMI percentile at Wave 2. We hypothesized that maternal BE would be associated with responses to children’s negative emotions at Wave 1, which would predict use of non-responsive feeding practices at Wave 2, which in turn would be associated with higher child BMI percentile at Wave 2 (Fig. 2).

## Methods

### Study design, Recruitment, & Procedures

Data for this study were drawn from the first two waves of the Synergistic Theory and Research on Obesity and Nutrition Group (STRONG) Kids program. The STRONG Kids Program is a 3-wave prospective panel study designed to assess transdisciplinary contributions to the development of childhood health behaviors and weight-related outcomes. At Wave 1, children were an average of 37 months old (*SD* = 6.94 months), and at Wave 2, children were an average of 57 months old (*SD* = 8.32 months). Parents of preschool-aged children were recruited from 31 licensed childcare centers in the Midwest. Parents self-reported their height and weight, and gave consent for researchers to measure their child’s height and weight at both waves. In addition, parents completed validated, self-report questionnaires in paper-and-pencil, or online format at each wave (for a detailed description of study design, recruitment, and follow-up see [46]). This study was approved by the Institutional Review Board at the authors’ university.

### Participants

Participants of the larger STRONG Kids Program included parents of preschool-aged children who consented to participation at both waves: *n* = 498 at Wave 1, and *n* = 299 at Wave 2. Sixteen fathers at Wave 1 and seven fathers at Wave 2 completed surveys, and 6 % of respondents in both waves were non-parent caregivers (e.g. relatives or other). Thus, fathers and non-parent caregivers were excluded from analyses due to limitations of power for subgroup analysis. The analytic sample consisted of cases with valid child biometric data and in which mothers completed both waves of the survey (*n =* 260). Descriptive statistics of model variables are presented in Table 1.

**Table 1:** Summary statistics and correlations among model variables

	1	2	3	4	5	6	7	8	9	10	11	12	13
1. Maternal BE	-												
2. W1 CCNES DR	.169**	-											
3. W1 CCNES PR	.060	.424**	-										
4. W1 CCNES MR	-.021	.249**	.570**	-									
5. W1 CCNES PFR	-.168*	-.213**	-.109	.000	-								
6. W1 CCNES EFR	-.157*	-.168*	-.171**	-.005	.606**	-							
7. W1 CCNES EER	-.103	-.192**	-.225**	.002	.688**	.487**	-						
8. W2 CFPQ BV	.036	-.157*	-.051	.014	.373**	.230**	.245**	-					
9. W2 CFPQ Inv	-.053	-.155*	-.081	.041	.281**	.186**	.122	.388**	-				
10. W2 CFPQ ER	.065	.165*	.201**	.142*	-.072	-.103	-.058	-.135*	-.055	-			
11. W2 CFPQ Mod	-.112	-.155*	-.076	.001	.347**	.276**	.211**	.620**	.419**	-.015	-		
12. W2 CFPQ Mon	.047	-.062	-.028	-.071	.106	.125	.058	.339**	.149*	-.158*	.325**	-	
13. W2 CFPQ Tch	-.062	-.101	-.043	.039	.303**	.278**	.163*	.554**	.432**	-.127*	.535**	.203**	-
14. W2 CFPQ ChC	.084	-.127	-.188**	-.021	.029	.008	.090	-.064	.029	.097	-.081	-.141*	.042
15. W2 CFPQ FR	.111	.249**	.205**	.125	-.037	-.059	-.003	-.028	-.045	.306**	-.089	-.034	-.103
16. W2 CFPQ PTE	-.061	.229**	.190**	.166*	-.028	-.112	.030	-.001	.027	.183**	.111	.055	-.056
17. W2 CFPQ RH	.089	.151*	.065	-.018	-.009	.001	-.071	.092	.050	.081	.143*	.127*	.090
18. W2 CFPQ RW	.038	.069	.160*	.082	-.138*	-.093	-.070	.010	-.011	.226	.077	.356**	.031
19. W2 CFPQ Env	-.031	-.155*	-.044	.002	.142*	.084	.129*	.461**	.312**	-.163*	.469**	.303**	.396**
20. W1 PBMI	.213**	-.024	.015	.038	-.171*	-.024	-.089	-.106	-.142*	.088	-.193**	-.062	-.134*
21. W2 PBMI	.239**	-.019	-.024	-.027	-.125	.002	-.062	-.067	-.121	-.130*	-.214**	-.065	-.144*
22. W1 CBMIP	-.121	-.094	-.064	.005	.010	.065	.059	.006	-.024	-.065	-.028	.003	-.006
23. W2 CBMIP	-.043	.026	.020	-.014	.012	-.002	.009	.020	-.090	-.106	-.007	.035	-.034
24. W1 Depress	.279**	.251**	.139*	.143*	-.204**	-.146	-.131*	-.133*	-.153*	.051	-.206**	-.126*	-.200**
25. W1 Anxiety	.106	.075	.075	.169*	-.041	-.011	-.022	.011	.105	.046	.005	-.070	.052
Mean	.2654	2.561	2.021	2.300	5.847	5.135	5.921	4.375	3.112	1.400	3.752	4.132	3.962
SD	.846	.637	.668	.736	.684	1.120	.716	.597	.789	.467	.875	.884	.742
Range	5.00	3.42	3.50	4.08	3.67	4.42	3.50	2.75	4.00	3.00	4.00	4.00	3.00
N	260	234	231	234	234	239	243	247	249	253	247	250	251
	14	15	16	17	18	19	20	21	22	23	24	25	
1. Maternal BE													
2. W1 CCNES DR													
3. W1 CCNES PR													
4. W1 CCNES MR													
5. W1 CCNES PFR													
6. W1 CCNES EFR													
7. W1 CCNES EER													
8. W2 CFPQ BV													
9. W2 CFPQ Inv													
10. W2 CFPQ ER													
11. W2 CFPQ Mod													
12. W2 CFPQ Mon													
13. W2 CFPQ Tch													
14. W2 CFPQ ChC	-												
15. W2 CFPQ FR	-.040	-											
16. W2 CFPQ PTE	-.211**	.228**	-										
17. W2 CFPQ RH	.072	.254**	.136*	-									
18. W2 CFPQ RW	-.038	.134*	.024	.324**	-								
19. W2 CFPQ Env	.016	-.257**	-.151*	-.109	.043	-							
20. W1 PBMI	.059	-.129*	-.078	-.119	-.078	-.116	-						
21. W2 PBMI	.046	-.122	-.093	-.078	-.077	-.124	.898**	-					
22. W1 CBMIP	-.161*	-.095	-.200**	.008	.151*	.014	.154*	.216**	-				
23. W2 CBMIP	-.112	-.065	-.200**	.046	.173*	-.014	.167*	.218**	.759**	-			
24. W1 Depress	.035	.085	.066	.139*	.006	-.155*	.189**	.230**	.030	.078	-		
25. W1 Anxiety	.067	.029	.023	.126	.001	-.091	.078	.078	.032	.020	.327**	-	
Mean	2.400	2.038	2.453	2.752	1.594	3.884	26.927	27.591	61.566	62.228	4.340	2.619	
SD	.650	.812	.849	.976	.533	.584	6.777	7.597	26.087	29.479	5.543	3.539	
Range	3.80	4.00	4.00	4.00	3.13	3.00	37.39	40.80	99.72	99.94	36.00	24.00	
N	249	249	247	247	240	249	246	256	227	237	253	252	

*Note. BE* Binge eating, *W*1 Wave 1, *CCNES* Coping with Children’s Negative Emotions Scale, *DR* Distress Responses, *PR* Punitive Responses, *MR* Minimizing Responses, *PFR* Problem-Focused Responses, *EFR* Emotion-Focused Responses, *EER* Expressive Encouragement Responses, *W*2 Wave 2, *CFPQ* Comprehensive Feeding Practices Questionnaire, *BV* Balance and Variety, *Inv* Involvement, *ER* Emotion Regulation, *Mod* Modeling, *Mon* Monitoring, *Tch* Teaching about nutrition, *ChC* Child Control, *FR* Food as Reward, *PTE* Pressure to Eat, *RH* Restriction for health, *RW* Restriction for weight control, *Env* Environment, *W*1 Wave 1, *PBMI* Parent Body Mass Index, *W*2 Wave 2, *CBMIP* Child Body Mass Index Percentile

*p < .05, **p < .01

### Measures

#### Demographics

Mothers self-reported age, race/ethnicity, height, weight, educational attainment, marital status, and income at Wave 1. Parent BMI was computed from self-reported height and weight [47] and was modeled as a continuous variable. These variables were included as control variables since they have been linked to childhood obesity in prior literature [48].

#### Child Body Mass Index [BMI]

Child BMI percentile at Wave 2 served as the outcome variable of interest in the second model (Fig. 2). Height and weight were directly measured at Wave 1 and at Wave 2 by trained research assistants, using standardized procedures [46]. Child height and weight were measured using a stadiometer and a digital scale. In order to establish reliability, two measurements were taken at each time point by different research assistants. Weight measurements were correlated at 1.00, and height measurements were correlated at 0.997 [46]. Child body mass index (BMI) percentile was computed using national guidelines based on age and sex [49]. Child BMI percentile was computed as a continuous variable. When examining effects on child BMI percentile at Wave 2, child BMI percentile at Wave 1 was modeled as a control variable.

#### Maternal binge eating frequency

Maternal binge eating (BE) episode frequency was assessed using the Eating Disorder Diagnostic Scale [EDDS] at Wave 1 when children were an average of 37 months old [50, 51]. The items in the EDDS allow for assessment of full- and subthreshold eating disorders using DSM-5 diagnostic criteria [27], and the EDDS has been found to have good internal consistency (*α* = .89), high test-retest reliability (*r* = .87), and adequate criterion validity (*k* = .74) in comparison to a diagnostic interview [51]. A binge eating frequency index was constructed using criteria consistent with DSM-5. Participants were first coded as binge eaters if they reported eating an unusually large amount of food with loss of control, and no compensatory behaviors (e.g., purging, laxative abuse) over the previous three months. If binge eating was reported, they also reported the number of binge episodes per week over the past three months. Then, we constructed an index based on the number of BE episodes that participants reported per week over the previous three months. The index ranged from 0 to 5, with zero assigned to those who were not coded as binge eaters (*n* = 226), and a score from 1 to 5 assigned depending on the number of BE episodes per week participants reported. A score of 5 was assigned when a participant reported five or more episodes per week. A score of 0.5 was assigned when individuals reported behaviors that met above criteria for BE, but did not report weekly BE episodes over the prior three months (*n* = 6). The index was modeled as a continuous independent variable in final analyses.

#### Mothers’ responses to children’s negative emotions

Maternal responses to children’s negative emotions were assessed at Wave 1, using the Coping with Children’s Negative Emotions Scale (CCNES; [40, 52]). The CCNES consists of twelve vignettes with six questions about a potential responses to children’s negative emotion in each vignette, yielding 72 items with good internal reliability (α = .86) [40]. Parents are asked to rate the likelihood of using each response, on a Likert scale from 1 (*very unlikely*) to 7 (*very likely*). Six theoretically distinct subscales describing various responses that parents have when their children express negative affect can be derived from the CCNES: Problem-Focused Responses (PFR), Emotion-Focused Responses (EFR), Expressive Encouragement Responses (EER), Punitive Responses (PR), Minimizing Responses (MR), and Distress Responses (DR). Subscale internal reliability in the present study ranged from *α =* .74 to .90, except for Distress responses (*α = .*61). Cronbach’s alphas in this study are comparable to prior studies [53, 54]. Each subscale was modeled as a continuous mediator variable; in the first model, the CCNES subscale was modeled as the only mediator (Fig. 1), and in the second model as Mediator 1 (Fig. 2).

#### Feeding practices

Maternal feeding practices served as the outcome variable in the first model (Fig. 1), and served as Mediator 2 in the second model (Fig. 2). Maternal feeding practices were assessed at Wave 1 and Wave 2, using the Comprehensive Feeding Practices Questionnaire (CFPQ; [55]). The CFPQ consists of 49 items, yielding 12 subscales regarding types of feeding practices used: Child Control (5 items) Emotion Regulation (3 items), Balance/Variety (4 items), Environment (4 items), Food as Reward (3 items), Involvement (3 items), Modeling (4 items), Monitoring (4 items), Pressure (4 items), Restriction for health (4 items), Restriction for weight control (8 items), Teaching about nutrition (3 items). Subscale internal reliability in the present study ranged from *α =* .59 to .90, which is comparable to previously reported Cronbach’s alpha statistics for these subscales (*a =* .58 to .81) [56]. For each item, mothers were asked to indicate how often they engaged in a feeding practice on a Likert scale from 1 (*Never*) to 5 (*Always*). Each subscale was modeled as a continuous variable.

#### Maternal depressive and anxiety symptoms

Maternal depressive and anxiety symptoms were measured at Wave 1, using the short-form of the Depression, Anxiety, and Stress Scale (DASS-21; [56]). Depressive (*α =* .84) and anxiety (*α =* .56) symptoms were measured with 7-item subscales for each, with internal reliability for depressive symptoms in the present study comparable to reports in extant literature (*α =* .88) [56]. Mothers self-reported the degree to which each item applied to them over the past week, from 0 (*Never*) to 3 (*Almost always*). Depressive and anxiety symptoms were used as continuous control variables.

### Statistical analysis

Data cleaning, descriptive, and bivariate analyses were conducted with SPSS Version 22.0 and mediation analyses were performed with Mplus version 7.4 [57, 58]. Due to non-normal distributions in model variables, we used Spearman’s correlations to assess initial associations between variables. In order to account for loss to follow-up bias, we conducted missing value analyses to identify any patterns in the data, and found evidence to suggest that data were not missing in a systematic pattern (MCAR *X*^*2*^*[df] =* 33510.647 [161294], *p =* 1.00). There was also evidence to suggest that data in the analytic sample (*n* = 260) were not missing in a systematic pattern (Little’s MCAR *X*^*2*^*[df] =* 57697.52 [61686], *p =* 1.00), and model variables had between 1.0 and 12.7 % missing data from the sample. Therefore, full information maximum-likelihood (FIML) estimation procedures were used to account for missingness in final analyses [59]. Because standard FIML estimation is slightly sensitive to non-normality [60], all analyses were run using both standard and robust FIML procedures to ensure valid estimates. There were no differences between results found by standard and robust FIML procedures, therefore only results using standard FIML procedures are presented. Although tests of statistical significance—as used in robust FIML procedures—are useful for assessing whether a relationship does or does not exist, confidence intervals provide more information about the magnitude of associations, and are therefore presented in tables and text [61, 62].

To test hypothesis 1 (Fig. 1) and hypothesis 2 (Fig. 2), we conducted simple and serial mediation analyses using Mplus.

## Results

### Sample characteristics

Of the (*n* = 260) mothers in the STRONG Kids sample, most identified as White (*n* = 203, 78.1 %), followed by Black or African American (*n* = 25, 9.6 %), Asian (*n* = 18, 6.9 %), and Hispanic/Latina (*n* = 14, 5.4 %). Participants were well educated, with most having least some college (*n* = 68, 26.2 %), graduated from college (*n* = 87, 33.5 %), or post-graduate work (*n* = 89, 34.2 %), likely due to the geographic location of the panel study, which required proximity to a large Midwestern university. Nevertheless, there was diversity in reported household income: 16 % (*n* = 42) made less than $24,999, 54.2 % made between $25,000 and $99,999, and 24.6 % (*n* = 64) made more than $100,000. Most (*n* = 195, 75 %) mothers were married, some were single (*n* = 42, 16.2 %) or co-habiting (*n* = 12, 4.6 %), and few were separated (*n* = 4, 1.5 %), divorced (*n* = 3, 1.2 %), or widowed (*n* = 2, 0.8 %). The mean BE index score among the full analytic sample of mothers was lower (*M* = .26, *SE* = .85) than the mean score among the sample of mothers who reported binge eating (*n* = 34, *M* = 2.03, *SE* = 1.39). Finally, of children, 53.1 % (*n* = 138) were female, and 45.8 % (*n* = 119) were male. At Wave 1, 19.6 % (*n* = 51) of children, and at Wave 2, 26.9 % (*n* = 70) of children were overweight or obese. Descriptive statistics and bivariate associations between model variables are presented in Table 1.

### Bivariate analyses

Maternal BE was positively associated with Distress and negatively associated with Problem-Focused and Emotion-Focused responses, but no other CCNES subscales. Maternal BE was also positively associated with parent BMI and depressive symptoms. It is important to note that bivariate associations between independent and dependent variables are not a prerequisite for modern tests of direct and indirect effects [62]. Rather, independent variables must be associated with mediators, and mediators must be associated with dependent variables, in order for test of indirect effects to be logically valid. In that vein, Distress responses on the CCNES were positively associated with Emotion Regulation, Food as Reward, Pressure to Eat, and Restriction for Health feeding practices, and negatively associated with Balance/Variety, Involvement, Modeling, and Environmental feeding practices. Problem-focused responses on the CCNES were positively associated with Balance/Variety, Involvement, Modeling, Teaching about Nutrition, and Environmental feeding practices, and negatively associated with Restriction for Weight Control feeding practices. Emotion-focused responses on the CCNES were positively associated with Balance/Variety, Involvement, Modeling, and Teaching about Nutrition feeding practices (Table 1).

Associations between model variables and potential covariates were also analyzed. Parent BMI was related to maternal BE, Problem-Focused Responses, Involvement, Modeling, Teaching about Nutrition, and Food Reward. Parent age was related to Pressure to Eat feeding practices; child age was related to Problem-focused and Emotion-focused responses, and to Involvement feeding practices. Finally, Asian mothers were slightly more likely than white mothers to use Pressure to Eat (*F [df]* = 2.607[5], *p* =. 026) and Restriction for Weight (*F [df]* = 2.538[5], *p* =. 029) feeding practices. Therefore, parent BMI, parent age, child age, maternal depression and race were included as covariates in relevant analyses.

### Mediation Analyses

We tested two models, the first of which posited that frequency of maternal BE at Wave 1 would predict maternal reports of non-responsive feeding practices at Wave 2 directly, and indirectly through mothers’ unsupportive responses to children’s negative emotions (Fig. 1). We found partial support for this hypothesis, statistically controlling for covariates (Table 2). Maternal BE predicted report of less frequent use of Balance/Variety and Involvement, and more use of Emotion Regulation, Food as Reward, Pressure to Eat, and Restriction for Health feeding practices indirectly through its effect on Distress Responses to children’s negative emotions. Maternal BE also directly predicted Restriction for Health and Pressure to Eat feeding practices, beyond the effects of Distress Responses, but no other direct effect pathways were significant. Maternal BE predicted less frequent use of Balance/Variety and Modeling feeding practices, indirectly through the reduction of Emotion-focused Responses. Similarly, maternal BE predicted less frequent use of Involvement feeding practices, indirectly through the reduction of Problem-focused Responses.

**Table 2:** Direct effects of maternal binge eating (BE) on feeding practices (CFPQ), and indirect effects of BE on feeding practices through responses to child negative emotion (CCNES) using bias-corrected bootstrapped confidence intervals (CI) and full-information maximum likelihood estimation ^a,b^

	Direct effect		Indirect effect		Total effect	
	BE→CFPQ		BE→CCNES→CFPQ			
IV: Maternal BE	B (SE)	95 % CI	B (SE)	95 % CI	B (SE)	95 % CI
M: CCNES Distress Responses						
DV: CFPQ Emotion regulation	.015 (.035)	(-.051, .084)	.016 (.011)*	(.003, .050)	.031 (.032)	(-.026, .096)
DV: CFPQ Food reward	.079 (.078)	(-.079, .218)	.031 (.016)*	(.010, .088)	.110 (.081)	(-.044, .267)
DV: CFPQ Restriction for health	.130 (.078)*	(.012, .290)	.039 (.020)*	(.013, .105)	.169 (.082)*	(.046, .347)
DV: CFPQ Pressure to eat	-.114 (.058)*	(-.263, -.026)	.024 (.017)*	(.003, .069)	-.090 (.051)*	(-.214, -.008)
DV: CFPQ Balance/variety	.052 (.044)	(-.032, .092)	-.016 (.012)*	(-.049, -.004)	.036 (.040)	(-.049, .107)
DV: CFPQ Involvement	.024 (.060)	(-.095, .143)	-.023 (.016)*	(-.083, -.003)	.001 (.062)	(-.122, .120)
M: CCNES Problem-focused Responses						
DV: CFPQ Balance/variety	.063 (.040)	(-.004, .154)	-.030 (.026)	(-.107, .006)	.033 (.036)	(-.046, .097)
DV: CFPQ Involvement	.037 (.060)	(-.072, .166)	-.029 (.021)*	(-.100, -.003)	.008 (.063)	(-.117, .128)
DV: CFPQ Modeling	-.043 (.061)	(-.163, .078)	-.034 (.027)	(-.112, .002)	-.078 (.067)	(-.112, .002)
M: CCNES Emotion-focused Responses						
DV: CFPQ Balance/variety	.065 (.043)	(-.009, .157)	-.033 (.018)*	(-.094, -.010)	.032 (.038)	(-.045, .104)
DV: CFPQ Modeling	-.045 (.064)	(-.173, .080)	-.034 (.020)*	(-.096, -.009)	-.079 (.065)	(-.214, .044)

*Note. IV* Independent Variable, *M* Mediator, *DV* Dependent Variable, *CFPQ* Comprehensive Feeding Practices Questionnaire, *CCNES* Coping with Children’s Negative Emotions Scale, *BE* Binge eating

^a^All models controlled for maternal BMI at Wave 1 and 2, and change in feeding practices from Wave 1 to Wave 2. Pressure to Eat feeding practices were also related to parent age, child age, child BMI percentile, and parent race/ethnicity and so these were included as additional controls in analyses estimating effects on Pressure to Eat only. Only pathways in which the IV was associated with the mediator, and the mediator was associated with the DV were tested, in accordance with criteria for testing mediation (Hayes et al., 2013)

^b^In order to account for missingness on exogenous covariates, all control variables were brought into the model in Mplus. There were few differences in results between models with control variables and without control variables in the model. Therefore, conservative findings with covariates in the model and no missingness are presented

The second hypothesis was that maternal BE at Wave 1 would predict higher child BMI percentile at Wave 2 indirectly through mothers’ report of using more unsupportive responses to children’s negative emotions (Fig. 2). We did find support for this hypothesis: maternal BE predicted higher child BMI percentile indirectly through mothers’ report of using more unsupportive responses to negative emotions and through certain unresponsive feeding practices (Table 3, Additional file 1: Table S1). The pathways from maternal BE to Food as Reward, indirectly through Distress responses to children’s negative emotions, were significant. Maternal BE also predicted higher child BMI percentile through the specific indirect effect of Distress Responses, independently of Balance/Variety, Pressure to Eat, Involvement, and Emotion Regulation feeding practices. No other serial mediation pathways were significant (Additional file 1: Table S1).

**Table 3:** Unstandardized direct and indirect effects of Wave 1 maternal binge eating (BE) frequency, Wave 1 Distress responses to children’s negative emotion (CCNES), and Wave 2 feeding practices (CFPQ) on child BMI percentile at Wave 2 ^a,b^

	B (SE)	95 % CI
(IV) Maternal BE→ (M1) CCNES Distress→ (M2) CFPQ Balance/Variety		
Total effect	.030 (1.312)	(-3.096, 1.797)
Direct effect	-.563 (1.286)	(-4.265, 1.014)
Indirect effect via CCNES Distress	.556 (.429)*	(.002, 1.901)
Indirect effect via Balance/Variety	.051 (.274)	(-.151, .770)
Specific indirect effect via CCNES Distress and Balance/Variety	-.015 (.047)	(-.237, .032)
Total indirect effect via CCNES Distress and Balance/Variety	.592 (.468)	(-4.265, 1.014)
(IV) Maternal BE→ (M1) CCNES Distress→ (M2) CFPQ Food Reward		
Total effect	.230 (1.357)	(-2.620, 2.418)
Direct effect	-.538 (1.280)	(-4.298, 1.219)
Indirect effect via CCNES Distress	.330 (.325)	(-.026, 1.729)
Indirect effect via Food Reward	.317 (.806)	(-.139, 1.498)
Specific indirect effect via CCNES Distress and Food Reward	.121 (.091)*	(.022, .510)
Total indirect effect via CCNES Distress and Food Reward	.768 (.523)*	(.125, 2.693)
(IV) Maternal BE→ (M1) CCNES Distress→ (M2) CFPQ Restriction-Health		
Total effect	.141 (1.306)	(-2.938, 2.025)
Direct effect	-.595 (1.290)	(-4.590, 1.024)
Indirect effect via CCNES Distress	.458 (.385)	(-.061, 1.547)
Indirect effect via Restriction-Health	.217 (.307)	(-.134, 1.333)
Specific indirect effect via CCNES Distress and Restriction-Health	.061(.074)	(-.014, .445)
Total indirect effect via CCNES Distress and Restriction-Health	.736 (.510)*	(.069, 2.493)
(IV) Maternal BE→ (M1) CCNES Distress→ (M2) CFPQ Pressure to Eat		
Total effect	.537 (1.344)	(-2.728, 2.425)
Direct effect	-.052 (1.358)	(-3.280, 1.654)
Indirect effect via CCNES Distress	.555 (.373)*	(.058, 1.746)
Indirect effect via Pressure to Eat	.043 (.256)	(-.227, .986)
Specific indirect effect via CCNES Distress and Pressure to Eat	-.009 (.057)	(-.241, .043)
Total indirect effect via CCNES Distress and Pressure to Eat	.589 (.439)*	(.058, 1.746)
(IV) Maternal BE→ (M1) CCNES Distress→ (M2) CFPQ Involvement		
Total effect	.072 (1.332)	(-2.995, 1.681)
Direct effect	-.408 (1.273)	(-3.990, 1.192)
Indirect effect via CCNES Distress	.475 (.442)	(-.114, 1.514)
Indirect effect via Involvement	-.029 (.136)	(-.667, .086)
Specific indirect effect via CCNES Distress and Involvement	.034 (.056)	(-.012, .378)
Total indirect effect via CCNES Distress and Involvement	.480 (.459)	(-.187, 1.519)
(IV) Maternal BE→ (M1) CCNES Distress→ (M2) CFPQ Emotion Regulation		
Total effect	.111 (1.366)	(-3.067, 2.002)
Direct effect	-.335 (1.281)	(-3.787, 1.242)
Indirect effect via CCNES Distress	.541 (.420)*	(.029, 1.890)
Indirect effect via Emotion Regulation	-.048 (.195)	(-.567, .229)
Specific indirect effect via CCNES Distress and Emotion Regulation	-.048 (.061)	(-.253, .015)
Total indirect effect via CCNES Distress and Emotion Regulation	.445 (.469)	(-.203, 1.894)

*Note. IV* Independent Variable, *M*1 Mediator 1, *M*2 Mediator 2, *DV* Dependent Variable, *CFPQ* Comprehensive Feeding Practices Questionnaire, *CCNES* Coping with Children’s Negative Emotions Scale

^a^All analyses adjusted for child BMI percentile at Wave 1, maternal BMI at wave 1 and 2, and change in feeding practices from Wave 1 to Wave 2. Pressure to Eat feeding practices were also related to parent age, child age, child BMI percentile, and parent race/ethnicity and so these were included as additional controls in analyses estimating effects on Pressure to Eat only

^b^In order to account for missingness on exogenous covariates, all control variables were brought into the model in Mplus. There were few differences in results between models with control variables and without control variables in the model. Therefore, conservative findings with covariates in the model and no missingness are presented

## Discussion

This study provides support for the hypotheses that emotional responsiveness mediates the association between maternal binge eating (BE) and feeding practices, and that emotional responsiveness may also influence child weight. Our main findings were as follows.

First, maternal BE was significantly related to use of unsupportive emotional response strategies—specifically Distress Responses—at Wave 1. In the eating disorder literature, it is well established that emotion regulation may be compromised among people with BED [63], and that people with BED utilize less adaptive emotion regulation strategies than healthy controls [64]. Maternal emotion regulation strategies are associated with maternal emotional response strategies and child emotion regulation [65], so it is not surprising that our findings revealed a significant association between BE and maternal reports of unsupportive emotional response strategies. This study adds to extant literature suggesting that energy-intake self-regulation and emotion-related self-regulation are linked processes. Moreover, to the best of our knowledge, this study is the first to find longitudinal associations between greater frequency of maternal BE—an example of dysregulated eating behavior—and unsupportive responses to their children’s negative emotions.

Second, use of Distress responses placed mothers with BE at risk for using more nonresponsive feeding practices—specifically Emotion Regulation, Food as Reward, Pressure to Eat, and Restriction for Health—two years later. Distress responses involve focus on the parent’s discomfort and negative affect, rather than on alleviating the child’s negative emotion [10, 11, 39, 40]. We interpret these findings to suggest that a parent who struggles to focus on their child’s emotional cues may also struggle to focus on their child’s hunger and satiety cues, especially if regulation around food intake is already compromised. Future research should explore whether supportive emotional responses predict use of fewer nonresponsive feeding practices among mothers with more typical eating behaviors.

Third, maternal binge eating was linked to higher child BMI percentile at Wave 2 (controlling for BMI percentile at Wave 1), indirectly through maternal use of Distress responses to children’s negative emotions, and use of Food as a reward feeding practices. There are a few possible interpretations of these findings. On one hand, it is possible that mothers with BE would provide food as a reward for good behavior since this may be more valuable or adaptive for their relationship with their child while they cope with psychological distress. On the other hand, it is also possible that food is a rewarding stimulus to mothers with BE [66]; this reward response may be apparent in mothers’ use of Food as a reward for good behavior with their children. Prior studies have also found associations between feeding responsiveness and overweight among 6-7-year old children [67], and 12-month old infants [68]. Thus, feeding practices and their degree of responsiveness may be a mechanism for the association between emotional responsiveness and child weight outcomes.

Unexpectedly, maternal BE also predicted higher child BMI percentile indirectly through Distress responses, independently of Balance/Variety, Pressure to Eat, and Emotion Regulation feeding practices. Conceptual scholarship has repeatedly called for examination of how emotion regulation may affect the intergenerational transmission of eating behavior and weight [18, 21]. To the best of our knowledge, this is the first study to suggest empirically that emotion responsiveness may be independently important for understanding weight outcomes among the children of mothers who binge eat.

No other feeding practices or emotional responses yielded a significant specific indirect effect for the specified pathways, which could be a reflection either of the sensitivity of the measures, or of the nature of the relationship. If it is the latter, mothers who binge eat may be overloaded with stress, and may struggle to use supportive emotional responses due to the psychological distress that often accompanies disordered eating behaviors. If it is the former, observational or lab-based assessments of emotional responsiveness, in addition to self-report measures, are needed.

These findings are theoretically consistent with Attachment Theory, which provides a useful framework for examining how maternal-child interactions could influence feeding and child weight outcomes [24, 43, 69]. Attachment insecurity has been previously linked to unhealthful food consumption in children, indirectly through unsupportive responses to negative emotion [24]. Attachment insecurity reflects patterns of interaction that yield an expectation that a child’s signals of distress or pleasure will be met with non-responsiveness, insensitive responsiveness, or inconsistent responsiveness from a parent. In this study, we found further evidence to suggest that patterns in maternal responsiveness may have implications for children’s health.

Although this study’s results are novel and point to promising avenues of future research, a few limitations should be highlighted. Data are all self-report (except child BMI percentile), and so may be susceptible to reporting bias. Parent weight in particular may be vulnerable to underreporting. Thus, we recommend that future research validate these findings with observations. Although we discuss supportive/responsive and unsupportive/non-responsive emotional response strategies and feeding practices, respectively, it is important to note that these distinctions are conceptual. The feeding practices identified as “non-responsive” have been characterized as such by previous literature [6, 8, 9], and characterization of emotional responses as unsupportive/supportive is similarly present in prior literature [24]. We narrowed our sample to mothers who completed surveys at both waves and conducted conservative analyses to examine and account for missing data within the analytic and the full sample, but we acknowledge that bias from loss to follow-up may still be present, and that data on fathers is critical to incorporate in future studies. Finally, our sample of mothers who engaged in BE was small (*n* = 36), and embedded within a convenience sample that was well educated, over 75 % White with limited representation from other ethnic groups, and from a specific geographic region, limiting generalizability. Nevertheless, this study contributes novel findings to the literature about maternal BE, and provides a model for how maternal eating behavior may affect feeding practices in the family and children’s weight status.

## Conclusions

Findings from this study have implications for research and practice. We demonstrated the utility of considering emotional responsiveness when estimating risk for feeding responsiveness and childhood obesity. Moreover, we found evidence to suggest that emotional responsiveness may be independently important for estimating obesity risk among children of mothers who engage in binge eating, beyond the effects of feeding practices. Interventions focused on modifying parent feeding practices rely mostly on disseminating information about healthful feeding [70, 71], with limited effects on child weight outcomes (see [71] for a systematic review). Interventions modifying maternal emotion socialization practices show promise for improving both maternal emotion socialization and child emotion regulation [72, 73]. Thus, it may be fruitful for future research to closely examine prospective relationships between emotion responsiveness, feeding responsiveness, and child weight.

In sum, in a longitudinal study of Midwestern mothers and their preschool-age children, maternal binge eating predicted reported increase of nonresponsive feeding practices through more unsupportive responses to children’s negative emotion. Moreover, we found that maternal binge eating at Wave 1 predicted increased child BMI at Wave 2 through reported increase of certain negative emotions and feeding patterns. These findings suggest that that the roles of maternal emotional and feeding responsiveness may be intertwined, and that both influence weight outcomes among children of mothers with binge eating.

## Abbreviations

BE, Binge eating; BED, Binge eating disorder; BMI, Body mass index; CCNES, Copying with Children’s Negative Emotion Scale; CFPQ, Comprehensive Feeding Practices Questionnaire; DASS-21, Depression, Anxiety, and Stress Scale; DSM-5, Diagnostic and Statistical Manual of Mental Disorders 5th edition; EDDS, Eating Disorder Diagnostic Scale

## Additional file

Additional file 1: Table S1. Unstandardized direct and indirect effects of Wave 1 maternal binge eating (BE) frequency, Wave 1 responses to children’s negative emotion (CCNES), and Wave 2 feeding practices (CFPQ) on child BMI percentile at Wave 2. (DOCX 16 kb)
