# Supplementary material for: Eating, feeding, and feeling: emotional responsiveness mediates longitudinal associations between maternal binge eating, feeding practices, and child weight
Source: Int J Behav Nutr Phys Act. 2016 Aug 2;13:89. doi: 10.1186/s12966-016-0415-5 (PMC4971716; doi:10.1186/s12966-016-0415-5)
Supplement: Additional file 1: Table S1. — Unstandardized direct and indirect effects of Wave 1 maternal binge eating (BE) frequency, Wave 1 responses to children’s negative emotion (CCNES), and Wave 2 feeding practices (CFPQ) on child BMI percentile at Wave 2. (DOCX 16 kb) [file 12966_2016_415_MOESM1_ESM.docx]

| Additional file 1: Table S1. Unstandardized direct and indirect effects of Wave 1 maternal binge eating (BE) frequency, Wave 1 responses to children’s negative emotion (CCNES), and Wave 2 feeding practices (CFPQ) on child BMI percentile at Wave 2. ^1,2^ | | | |
| --- | --- | --- | --- |
|  | B (SE) |  | 95% CI |
| **(IV) Maternal BE🡪 (M1) CCNES Problem-Focused Responses (PFR) 🡪 (M2) CFPQ Balance/Variety** | | | |
| Total effect | .167 (1.338) |  | (-3.015, 2.070) |
| Direct effect | .049 (1.322) |  | (-3.141, 1.771) |
| Indirect effect via CCNES PFR | .088 (.254) |  | (-.174, 1.042) |
| Indirect effect via CFPQ Balance/Variety | .054 (.241) |  | (-.209, .851) |
| Specific indirect effect via CCNES PFR and Balance/Variety | -.025 (.114) |  | (-.565, .061) |
| Total indirect effect via CCNES PFR and Balance/Variety | .118 (.338) |  | (-.305, 1.273) |
| **(IV) Maternal BE🡪 (M1) CCNES PFR 🡪 (M2) CFPQ Involvement** | | | |
| Total effect | .219 (1.329) |  | (-2.999, 1.911) |
| Direct effect | .183 (1.308) |  | (-2.935, 1.925) |
| Indirect effect via PFR | .045 (.234) |  | (-.461, .493) |
| Indirect effect via Involvement | -.061 (.135) |  | (-1.170, .022) |
| Specific indirect effect via PFR and Involvement | .052 (.065)* |  | (.003, .488) |
| Total indirect effect via CCNES PFR and Involvement | .036 (.262) |  | (-2.999, 1.911) |
| **(IV) Maternal BE🡪 (M1) CCNES PFR 🡪 (M2) CFPQ Modeling** | | | |
| Total effect | .348 (1.322) |  | (-2.896, 2.057) |
| Direct effect | .301 (1.334) |  | (-2.929, 2.074) |
| Indirect effect via CCNES PFR | .099 (.281) |  | (-.306, .915) |
| Indirect effect via Modeling | -.027 (.137) |  | (-.537, .127) |
| Specific indirect effect via CCNES PFR and Modeling | -.025 (.084) |  | (-.314, .075) |
| Total indirect effect via CCNES PFR and Modeling | .047 (.287) |  | (-.500, .710) |
| **(IV) Maternal BE🡪 (M1) CCNES Emotion-Focused Responses (EFR) 🡪 (M2) CFPQ Balance/Variety** | | | |
| Total effect | .140 (1.352) |  | (-3.283, 1.944) |
| Direct effect | -.180 (1.308) |  | (-3.501, 1.506) |
| Indirect effect via CCNES EFR | .274 (.309) |  | (-.150, 1.069) |
| Indirect effect via Balance/Variety | .085 (.230) |  | (-.150, .954) |
| Specific indirect effect via CCNES EFR and Balance/Variety | -.039 (.092) |  | (-.440, .046) |
| Total indirect effect via CCNES EFR and Balance/Variety | .320 (.374) |  | (-.264, 1.288) |
| **(IV) Maternal BE🡪 (M1) CCNES EFR 🡪 (M2) CFPQ Modeling** | | | |
| Total effect | .306 (1.340) |  | (-3.104, 1.884) |
| Direct effect | .064 (1.306) |  | (-3.402, 1.724) |
| Indirect effect via CCNES EFR | .305 (.358) |  | (-.292, .987) |
| Indirect effect via Modeling | -.033 (.142) |  | (-.566, .129) |
| Specific indirect effect via CCNES EFR and Food Reward | -.030 (.069) |  | (-.231, .073) |
| Total indirect effect via CCNES EFR and Food Reward | .241 (.378) |  | (-.497, .926) |
| *Note*. IV=Independent Variable, M1=Mediator 1, M2=Mediator 2, DV=Dependent Variable, CFPQ = Comprehensive Feeding Practices Questionnaire, CCNES= Coping with Children’s Negative Emotions Scale.  ^1^All analyses adjusted for child BMI percentile at Wave 1, maternal BMI at wave 1 and 2, and change in feeding practices from Wave 1 to Wave 2.  ^2^In order to account for missingness on exogenous covariates, all control variables were brought into the model in Mplus. There were few differences in results between models with control variables and without control variables in the model. Therefore, conservative findings with covariates in the model and no missingness are presented. | | | |
